# Investigation of the Genotoxicological Profile of Aqueous *Betula pendula* Extracts

**DOI:** 10.3390/plants11202673

**Published:** 2022-10-11

**Authors:** Ioanna Efthimiou, Dimitris Vlastos, Vassilios Triantafyllidis, Antonios Eleftherianos, Maria Antonopoulou

**Affiliations:** 1 Department of Sustainable Agriculture, University of Patras, GR-30100 Agrinio, Greece; 2 Institute of Marine Biology, Biotechnology and Aquaculture, Hellenic Centre for Marine Research (HCMR), GR-19013 Anavyssos, Greece; 3 Department of Biology, Section of Genetics Cell Biology and Development, University of Patras, GR-26500 Patras, Greece; 4 Department of Food Science & Technology, University of Patras, GR-30100 Agrinio, Greece; 5 Akrokeramos Sewerage Laboratory, Athens Water Supply and Sewerage Company (EYDAP SA), GR-18755 Keratsini, Greece

**Keywords:** *Betula pendula*, cytotoxicity, genotoxicity, antigenotoxicity, human lymphocytes, cytokinesis block micronucleus assay

## Abstract

*Betula pendula* belongs to the *Betulaceae* family and is most common in the northern hemisphere. Various birch species have exhibited antimicrobial, antioxidant and anticancer properties. In the present study, we investigated the genotoxic and cytotoxic activity as well as the antigenotoxic potential against the mutagenic agent mitomycin-C (MMC) of two commercial products, i.e., a *Betula pendula* aqueous leaf extract product (BE) and a *Betula pendula* product containing aqueous extract of birch leaves at a percentage of 94% and lemon juice at a percentage of 6% (BP) using the cytokinesis block micronucleus (CBMN) assay. The most prevalent compounds and elements of BE and BP were identified using UHPLC-MS and ICP-MS/MS, respectively. All mixtures of BE with MMC demonstrated a decrease in the MN frequencies, with the lowest and highest concentrations inducing a statistically significant antigenotoxic activity. BP lacked genotoxic potential, while it was cytotoxic in all concentrations. Its mixtures with MMC demonstrated statistically significant antigenotoxic activity only at the lowest concentration. UHPLC-MS and ICP-MS/MS showed the presence of various elements and phytochemicals. Our results reveal antigenotoxic and cytotoxic potential of both BE and BP, while the variations observed could indicate the importance of the interactions among different natural products and/or their compounds.

## 1. Introduction

The use of natural products in medicinal applications has been known since ancient times. Their continuous investigation can expand the knowledge about their pharmacological and medicinal properties and explore their potential use for the treatment of many diseases.

*Betula pendula* belongs to the *Betulaceae* family and exhibits various pharmaceutical properties. The antimicrobial, antioxidant and anticancer activities of various birch species and their components are well-documented in the literature [1]. Moreover, the leaf extracts of *Betula pendula* have been widely used for the treatment of rheumatoid arthritis or osteoarthritis [2]. However, until now, the scientific research has been mainly focused on the identification of chemical constituents of the birch bark and the evaluation of their benefits [3,4,5], rather than those of the birch leaves. As far as lemon juice is concerned, it has been found to contain several beneficial constituents, including polyphenols, vitamins, minerals, dietary fiber and carotenoids that contribute to the prevention of chronic diseases such as diabetes, cardiovascular diseases and certain types of cancer [6,7].

The possible genotoxic and cytotoxic activity of two birch products: an aqueous leaf extract (BE) and a mixture of aqueous birch leaf extract and lemon juice (BP), as well as their antigenotoxic potential were evaluated. To achieve the goals of the study, the widely used cytokinesis block micronucleus (CBMN) assay in human lymphocytes cultures was selected and employed. The CBMN assay is a sensitive indicator, due to the potential induction of micronuclei (MN) in the cytoplasm of interphase human lymphocytes. The antigenotoxic effect of both birch products was studied against the genotoxic damage induced by mitomycin C (MMC). In the present study, the rationale, the experimental procedure and design are based on the preliminary results on the BE as published by Efthimiou et al. [8]. To move our work a step forward, we followed a multidisciplinary approach conducting duplicated experiments for both BE and BP to increase the statistical power of our results and to clarify their potential genotoxic, cytotoxic and antigenotoxic activities. Considering that in recent years the constituents of plant extracts have received a great deal of attention, the chemical composition of BE and BP with advanced mass spectrometry techniques, i.e., UHPLC-MS and ICP-MS/MS was also determined. 

## 2. Results

### 2.1. CBMN Assay in Human Lymphocytes In Vitro

Four different doses i.e., 0.5, 1, 2 and 5% (*v*/*v*) of the total culture volume of both birch products were studied. The same doses were also tested combined with MMC at a concentration of 0.05 μg mL^−1^ for BE and 0.5 μg mL^−1^ for BP in order to identify their antigenotoxic effect against the genotoxic damage induced by MMC. A treatment with 0.5, 1, 2 and 5% (*v*/*v*) of the BE did not induce MN as compared to the control. Similarly, the BP did not lead to MN induction in any concentration, with the exception of 5% which was too cytotoxic and therefore there were not enough binucleated (BN) cells available to count. Treatment with 0.05 and 0.5 μg mL^−1^ of MMC induced significant MN frequencies, as expected, compared to the control. A statistically significant decrease in MN frequency was found when 0.5 and 5% (*v*/*v*) of the BE treatment was given along with MMC, whereas only 0.5% (*v*/*v*) of BP-MMC treatment demonstrated similar results (Table 1 and Table 2).

The cytotoxic effect of the birch products and their mixtures with MMC was evaluated via the determination of CBPI. Regarding the cytotoxic index, statistically significant differences were detected between control cultures and the two highest doses of the BE as well as all the doses of the BP. Moreover, a statistically significant cytotoxic effect was observed between MMC and 1% (*v*/*v*) of BE with MMC as well as between MMC and all of its mixtures with the BP (Table 1 and Table 2).

### 2.2. UHPLC-MS Analysis

UHPLC-MS analysis was carried out to identify the main compounds of BE and BP. The proposed compounds along with their molecular ions [M-H]^-^ and their area percentage in BE (%A_BE_) and BP (%A_BP_) are presented in Table 3. The identification was based on the interpretation of their MS spectra as well as literature data. In BE quercetin, quercetin-*O*-hexuronide, quercetin-3-*O*-galactoside, quercetin-3-*O*-glucoside and sakuranetin were identified under the applied analytical conditions. The aforementioned compounds are in accordance with previous results [2]. Similarly, the BP contains all the aforementioned components found in BE, in addition to 5-p-coumaroylquinic acid, catechin and quercetin-3-*O*-arabinoside. A variety of compounds attributed to the lemon juice contained in the BP were also identified (Table 3), which have been previously mentioned by Spínola et al. [9] through HPLC-DAD-ESI-MS^n^ analysis.

### 2.3. ICP-MS/MS Analysis

The elements determined in the two birch products and their corresponding concentrations in mg L^−1^ are shown on Table 4. Phosphorus (P), silicon (Si), potassium (K) and aluminium (Al) were the major elements in BE, while potassium (K), phosphorus (P), magnesium (Mg) and calcium (Ca) were found at the highest concentrations in BP. The presence of lemon juice in BP may contribute to these differences. 

## 3. Discussion

Various experimental in vitro and/or in vivo test systems are used to investigate the bioactivity of natural products and plant derived compounds. One of the most used methods among the different types of well-established genotoxicity and cytotoxicity tests is the CBMN assay. The application of CBMN assay in human lymphocytes cultures is used to identify potential genotoxic and cytotoxic effects of plant derived compounds as well as to estimate their potential antigenotoxic activity against known genotoxic/mutagenic agents [10,11,12]. The CBMN assay is a multi-endpoint cytogenetic technique that enables measurement of several nuclear abnormalities such as structural/numerical chromosome aberrations and chromosome mal-segregation [13]. 

The bioactivity identified by an in vitro method, such as the CBMN assay, provides basic understanding of the potential genotoxic, cytotoxic and antigenotoxic profile of a plant derived extract in addition to examining its capability to constitute a suitable candidate for pharmaceutical applications [11]. In the present study, the genotoxic, cytotoxic and antigenotoxic effects of BE and BP were evaluated using the in vitro cytokinesis block micronucleus (CBMN) assay. 

### 3.1. Genotoxicity

The absence of genotoxicity showed by the BE could be explained by its main phenolic components such as quercetin derivatives. Quercetin, a naturally occurring flavonol, has been previously investigated for its genotoxic potential indicating lack of genotoxicity [14,15]. Regarding BP, the absence of genotoxic potential could be further explained by the presence of lemon juice constituents (Table 3). Limonene, the distinctive flavor component in lemon juice, is considered as a non-genotoxic carcinogen [16] even though mutagenicity assays showed negative results in *Salmonella* [17] and rats [18]. In previous research concerning the potential mutagenic and antigenotoxic activity of lemon juice, limonene and hesperidin, it was found that all substances were non mutagenic at the lowest concentration used in the wing spot test of *Drosophila* but lemon juice and limonene showed mutagenicity at the highest concentration [19]. Hesperidin showed lack of genotoxicity in the *Salmonella* TA98 assay with or without metabolic activation, as found by Van der Merwe et al. [20]. Limonene was not mutagenic in the Ames system using four strains of *Salmonella typhimurium* (TA98, TA100, TA1535 and TA1537) [17]. In addition to this, possible synergistic and/or antagonistic activities among the BP’s constituents could be attributed to the absence of genotoxic effects [21,22]. 

### 3.2. Antigenotoxicity

Taking into consideration the fact that compounds which reduce the damage caused by mutagenic agents are effective antigenotoxic/antimutagenic factors, we also investigated the potential antigenotoxic/antimutagenic effects of BE and BP against the mutagenic inducer mitomycin-C (MMC) in human lymphocytes. 

MMC is widely used as clastogen in both in vitro and in vivo test systems, as it exerts genotoxic and oxidative effects on several mammalian cells and animals. MMC is proposed as a positive control by the OECD protocol during the application of the CBMN assay [23,24,25]. MMC affects DNA synthesis by cross-linking the complementary strands found in DNA with absolute specificity and high efficiency for the CpG sequence. Moreover, MMC has clearly demonstrated its utility as an antitumor/chemotherapeutic agent and has been used in the systemic therapy of various types of cancer in combination with other drugs and references therein [26,27,28,29,30].

In our experimental test system, MMC was found to be genotoxic, inducing a statistically significant increase in MN and BNMN (Table 1 and Table 2). Based on our results, all the tested concentrations of BE co-treated with MMC led to a decrease in MN frequency which was statistically significant at the lowest and highest concentration, i.e., 0.5 and 5% (*v*/*v*) (Table 1). The observed induction of antigenotoxic potential could be related with BE’s main constituents, quercetin and its derivatives (Table 3). Ramos et al. [31] demonstrated that quercetin prevented DNA damage and possessed antiproliferative properties in human hepatoma cell line (HepG2) against *tert*-butyl hydroperoxide (*t*-BHP), while increasing the rate of DNA repair. In addition, quercetin exerted antigenotoxic effects in polychromatic erythrocytes of mouse peripheral blood against chromium trioxide [32]. Only the lowest BP concentration (0.5 % (*v*/*v*)) exerted statistically significant antigenotoxic activity (Table 2). Similar results have been previously reported by our research team on the case of Chios Mastic Water (CMW), an aqueous extract of the plant *Pistacia lentiscus var. Chia* [10], where the lowest dose of CMW induced the highest antigenotoxic activity. This is also corroborated by several other reports concerning different substances [19,33,34,35] leading to the possible conclusion that some of BP’s constituents might act as free radical scavengers at the lowest concentration and as pro-oxidants at the higher concentrations. Various studies concerning the antigenotoxic and protective potential of lemon juice and its constituents have been carried out. Higashimoto et al. [36] found a 36% mutagenicity-reducing activity of lemon juice against nitrite-treated 1-methyl-1,2,3,4-tetrahydro-carboline-3-carboxylic acid (MTCCA) using the TA100 strain of *Salmonella typhimurium*. The inhibitory capacity of hesperidin against the genotoxic effects of H_2_O_2_ in the imaginal discs of *Drosophila* was higher at the lowest concentration (55.5%) [19]. Kalpana et al. [37] found hesperidin produced radioprotection by effectively decreasing MN frequency, dicentric aberrations and comet attributes, and correlated this activity with the ability for ROS scavenging. Limonene inhibited the genotoxicity of H_2_O_2_, behaving as a reductor agent that would protect cells from H_2_O_2_-induced oxidative stress [38,39]. Costa and Nepomuceno [40] indicated that a mixture of vitamins and elements such as copper, selenium and zinc protects against the genotoxic effects of the chemotherapeutic free-radical generator Doxorubicin using the wing spot test in *Drosophila melanogaster*. 

### 3.3. Cytotoxicity

As far as the cytotoxicity is concerned, a significant decrease in CBPI values was observed at the two highest concentrations of BE, at 1 % (*v*/*v*) of BE with MMC and at all the concentrations of BP and BP-MMC mixtures (Table 1 and Table 2). The induction of cytotoxicity by the BE is in accordance with previous studies concerning various *Betula* species. The cytotoxic potency of the BP could be attributed to the synergistic effects of both the aqueous birch extract and the lemon juice constituents. Goun et al. [41] evaluated the activity of methylene chloride and methanol extracts of *Betula pendula* against leukemia and revealed their inhibitory effects on the growth of mouse leukemia cells (L1210). Additionally, the antiproliferative activity against B16 melanoma cell by *Betula pendula* fractions was reported by Calliste et al. [5]. Ju et al. [42] demonstrated that extracts of *Betula platyphylla* var. *japonica* induced apoptotic events in human promyelocytic leukemia (HL-60) cells. Lemon juice at percentages equal or greater than 30% was found to be cytotoxic towards Caco-2 cells (Caco-2 cell line is a continuous cell of heterogeneous human epithelial colorectal adenocarcinoma cells) [43]. Hesperidin, which is the major flavonoid in lemon, has been shown to prevent bone mass loss [44], chemically induced breast cancer [45], bladder cancer [46] and colon cancer [47,48,49] in animals. Furthermore, Lu et al. [50] found that limonene inhibits tumor growth and metastasis via apoptosis. Hesperidin and limonene exerted cytotoxic effects on HL60 cells. With respect to the cytotoxicity of limonene, Rabi and Bishayee [51] demonstrated the apoptotic effect of limonene in DU-145 prostate cancer cells but not in normal epithelial prostate PZ-HPV-7 cells. According to a recent study, both D- and L-limonene exerted antiviral activity against influenza A virus H1N1 [52]. In vivo assays for limonene are contradictory, as it seems to inhibit the appearance of liver and gastric tumors in mice [50,53] but Turner et al. [18] found that limonene induced kidney and bladder tumors in male rats.

### 3.4. UHPLC-MS and ICP-MS/MS Analysis

It is well known that the individual components of natural products are playing an important role in their bioactivity. In order to determine the presence of different phytochemicals in BE and BP, we carried out chemical analysis by using mass spectrometry techniques. 

The results from the UHPLC-MS analysis corroborate the above, since quercetin and its derivatives were identified as basic compounds of both birch products, while in the case of BP, additional components attributed to lemon juice contributed potentially to its antigenotoxic and cytotoxic activity. Furthermore, several elements were found via ICP-MS/MS, which have been mentioned by previous studies concerning birch sap [54] and lemon fruit [55]. However, variations in element content in BE and BP were observed. In general, this can be correlated with various factors such as plant species, plant age, soil characteristics, climate and implementation of agrotechnical methods. Moreover, the same plant species’ element content varies under different ecological conditions and geographic locations, while diverse species in the same ecosystem accumulate different amounts of microelements [56]. 

The biological importance of elements which are involved in fundamental processes in mammalian cells is under investigation and there is a plethora of publications in this topic. Considering that various disorders result and/or are connected with imbalances in bioactive elements, several therapeutic approaches in human diseases, including cancer, incorporate and propose the administration of different combinations of elements in parallel with conventional drugs [57,58,59].

Elements such as K, Ca, Fe, Mn, Cu, Mg and Zn (in small amounts) are of fundamental importance for human health, since deficiency of essential elements in the diet can result to several health disorders. Medicinal plant extracts are significant sources of such elements and can contribute to the dietary human requirements [56]. In general, micronutrients are indispensable to DNA metabolic pathways and certain micronutrients, such as Ca, Mg and Fe among others have been reported to play a critical role in cellular processes. Cu is considered as an essential trace element, serving as a cofactor for many enzymes in different biological processes. Zn is also an important micronutrient due to the prevalence of Zn-dependent enzymes in metabolic processes. [60]. Phosphorus is an important constituent of adenosine triphosphate (ATP), nucleic acids and phospholipids, whereas Mn activates several important enzyme systems among its other functions [61]. 

### 3.5. Natural Substances Interactions

Plants have been found to target pathogens via the combined activity of their constituents [62,63]. As a result, the beneficial effects of most natural substances could be due to the interactions and synergistic effects between the various components that co-exist [10,64,65,66]. Considering the results of studies showcasing that disease resistance is decreased against a combination of components rather than single compounds, it is highlighted that natural product mixtures could constitute very promising candidates for drugs and medicinal applications [67,68]. Thus, it is of paramount importance to investigate the safety and the efficacy of natural products and their mixtures shedding light to their potential synergistic, additive or antagonistic activity. In our study, the cytotoxicity of BP was more pronounced than that of BE. The combination of aqueous birch leaf extract and lemon juice in the case of BP could potentially lead to this result. In fact, quercetin, which was found to be present in both BE and BP, has been found to exert enhanced inhibitory activity against cancer cell proliferation when combined with curcumin [69,70]. Hesperetin and naringenin, whose glucosides hesperidin and narirutin were detected in BP, were used in combination and exhibited significant anticancer activity against human pancreatic cancer [71]. Liu et al. [72] demonstrated the synergistic effects of apigenin, whose glucoside Vitexin was present in BP, combined with metal ions against human cervical cancer Hela cells. Even though both BE and BP exhibited antigenotoxic activity, it was statistically significant in the lowest and highest concentrations of BE and only the lowest concentration of BP. Interestingly, it has been previously demonstrated that there were variations on the effect exhibited whether it be synergistic, additive or antagonistic depending on the dosage of the combined constituents [73]. 

### 3.6. Plant–Drug Interactions

It has been proven that a great number of diseases are treated more successfully through the administration of a combination of drugs [74]. Phytotherapy, i.e., the use of plants, herbs and natural products in general for the treatment of various ailments, has been gaining popularity these past decades. Combination of phytotherapy with conventional drugs could lead to enhanced effectiveness and reduction in the potential negative impacts [75]. MMC, which was used as a positive control in the present study, has been used as a drug against various tumors [76]. The increase in cytotoxic potential in two of the BE-MMC concentrations and all the concentrations of the BP-MMC mixtures indicated the potential enhancement of MMC’s activity in the presence of the two birch products which could be further studied and implemented for medicinal applications. In fact, the combination of the plant *Rauwolfia vomitoria* with anticancer drug carboplatin led to an increased chemosensitivity of ovarian cancer cells while inhibiting tumor growth in a mouse model with intraperitoneal metastasis [77]. Furthermore, the treatment of HT29 colon cancer cells with *Garcinia* benzophenones combined with different chemopreventive agents resulted in an enhanced ability of blocking cancer cell growth [78]. Synergistic antiproliferative effects were observed against pancreatic, breast and prostate cancer cells after being treated with a mixture of *Beta vulgaris* extract and doxorubicin [79]. 

Our findings are in accordance with our previous work [8], verifying the exerted cytotoxic and antigenotoxic effects of BE on human lymphocytes. Moreover, the importance of the combination of natural products and/or plant extracts is confirmed. 

## 4. Materials and Methods

### 4.1. Extracts

The aqueous leaf extract of *Betula pendula* was purchased from Abnoba GmbH (Betula folium D3 Abnoba, batch-no. 706 A41) [80] and the Birch juice product was purchased from Weleda [81]. 

### 4.2. Chemicals-Reagents 

All the chemical reagents used for CBMN assay as well as UHPLC-MS and ICP-MS/MS are reported in our previous works [8,12].

### 4.3. Ethics Statement

The CBMN assay using human lymphocytes was carried out in accordance with international bioethics criteria, after the permission/approval of the Research Ethics Committee of the University of Patras (Ref. No. 7682/6 June 2021). After obtaining the written informed consent four healthy, nonsmoking male individuals (less than 30 years), were used as blood donors to establish whole blood lymphocyte cultures. According to the donors’ declaration, they were not exposed to radiation, drug treatment or any viral infection in the recent past.

### 4.4. CBMN Assay in Human Lymphocytes In Vitro

The CBMN assay was performed according to standard procedures [25] with minor modifications. A full description of the assay can be found in our previous works [8,12,82]. Standard criteria were followed for the scoring of 2000 BN cells with preserved cytoplasm per concentration, so as to estimate the MN frequency [83,84]. Cytotoxicity was evaluated via the calculation of the cytokinesis block proliferation index (CBPI); 1000 cells with 1 (M1), 2 (M2), 3 (M3) and 4 (M4) nuclei were counted for each experimental point as previously mentioned [76]. CBPI is given by the equation:

CBPI = [M1 + 2M2 + 3(M3 + M4)]/N(1)

where N corresponds to the total number of cells.

### 4.5. Statistical Analysis

The statistical analysis was conducted according to previous work [10].

### 4.6. Preparation of Betula Pendula Products for UHPLC-MS and ICP-MS/MS

For UHPLC-MS analysis, the BE was directly used. For ICP-MS/MS analysis, 2 mL of the BE were diluted in 30 mL of an aqueous acid solution of 2.5 % *v*/*v* HNO_3_ and 0.5% *v*/*v* HCl. In the case of BP and for UHPLC-MS analysis, 2.5 mL of the product were gently heated at 40 °C up to total evaporation. After reaching room temperature the residue was dissolved with 7.5 mL of water (shortly vortexed). For ICP-MS/MS, 10 mL of the BP were gently heated at 40 °C up to total evaporation and the residue was allowed to reach room temperature. It was further dissolved in 30 mL of an aqueous acid solution of 2.5% *v*/*v* HNO_3_ and 0.5% *v*/*v* HCl (shortly vortexed).

### 4.7. UHPLC-MS Analysis

UHPLC-MS analysis was performed on the instrumentation reported previously [12]. The mobile phase was water with 0.1% formic acid (A) and acetonitrile (B) with a flow rate of 0.2 mL/min. The following gradient program was used: 14% B (0 min), 19% B (20 min), 95% B (22 min) 95% B (29 min), 14% B (32 min) and 14% B (35 min). The injection volume was 10 μL.

### 4.8. ICP-MS/MS Analysis

The elements were determined by an Agilent 8900 Triple Quadrupole ICP-MS/MS (Agilent Technologies, Japan) described in our previous work [12] and based on ISO 17294-2 testing protocols [85,86]. 

## 5. Conclusions

Our results reveal significant findings on the antigenotoxic and cytotoxic potential, of both BE and BP under the applied experimental conditions. All mixtures of BE and MMC demonstrated a decrease in the MN frequencies, with the concentrations of 0.5, and 5% (*v*/*v*) inducing a statistically significant antigenotoxic activity. BP did not increase the frequency of micronuclei (MN); however, it was found to be cytotoxic in all concentrations. The mixtures of BP and MMC did not show a decrease in the MN frequencies, except for the lowest concentration, which demonstrated statistically significant antigenotoxic activity. Therefore, both studied birch products revealed antigenotoxic and cytotoxic properties and their potential pharmaceutical and/or medicinal applications could be considered. It is noteworthy to mention that the differences observed between the activities exhibited by the two products could insinuate the importance of the interactions among different natural extracts and/or their various components. UHPLC-MS and ICP-MS/MS showed the presence of various elements and phytochemicals that justified the observed activities.

## Figures and Tables

**Table 1:** Frequencies of BNMN and MN as well as CBPI values in cultured human lymphocytes treated with BE, MMC (0.05 μg mL^−1^) and their mixtures.

Treatment	BNMN MF (‰ ± se)	MN MF (‰ ± se)	CBPI MF (‰ ± se)
Control	4.5 ± 0.5	4.5 ± 0.5	1.73 ± 0.13
0.5% (*v*/*v*) BE	4.0 ± 0.0	4.0 ± 0.0	1.72 ± 0.13
1% (*v*/*v*) BE	4.5 ± 1.5	4.5 ± 1.5	1.72 ± 0.12
2% (*v*/*v*) BE	3.5 ± 0.5	3.5 ± 0.5	1.60 ± 0.11 ^1^
5% (*v*/*v*) BE	4.0 ± 0.0	4.0 ± 0.0	1.57 ± 0.17 ^1^
0.05 μg mL^−1^ MMC	59.0 ± 6.0 ^1^	59.0 ± 6.0 ^1^	1.48 ± 0.02 ^1^
0.5% (*v*/*v*) BE + MMC (0.05 μg mL^−1^)	44.5 ± 3.5 ^1^^,a^	44.5 ± 3.5 ^1^^,a^	1.51 ± 0.06 ^1^
1% (*v*/*v*) BE + MMC (0.05 μg mL^−1^)	52.5 ± 1.5 ^1^	54.5 ± 1.5 ^1^	1.40 ± 0.01 ^1^^,a^
2% (*v*/*v*) BE + MMC (0.05 μg mL^−1^)	52.0 ± 10.0 ^1^	52.5 ± 10.5 ^1^	1.54 ± 0.12 ^1^
5% (*v*/*v*) BE + MMC (0.05 μg mL^−1^)	41.5 ± 4.5 ^1^^,a^	43.0 ± 5.0 ^1^^,a^	1.45 ± 0.07 ^1^

BNMN: micronucleated binucleated cells; MN: micronuclei; CBPI: cytokinesis block proliferation Index; BE: *Betula pendula* aqueous leaf extract product; MMC: mitomycin-C; MF (‰) ± se: mean frequencies (‰) ± standard error; MN were scored in 2000 binucleated lymphocytes per experimental point. ^1^ Significant difference compared to control at *p* < 0.001. ^a^ Significant difference compared to MMC at *p* < 0.05; G-test for BNMN and MN; *χ^2^* for CBPI.

**Table 2:** Frequencies of BNMN and MN as well as CBPI values in cultured human lymphocytes treated with BP, MMC (0.5 μg mL^−1^) and their mixtures.

Treatment	BNMN MF (‰ ± se)	MN MF (‰ ± se)	CBPI MF (‰ ± se)
Control	4.5 ± 0.5	4.5 ± 0.5	1.91 ± 0.03
0.5% (*v*/*v*) BP	3.5 ± 0.5	3.5 ± 0.5	1.62 ± 0.06 ^1^
1% (*v*/*v*) BP	4.0 ± 1.0	4.0 ± 1.0	1.60 ± 0.03 ^1^
2% (*v*/*v*) BP	4.5 ± 1.5	5.0 ± 1.0	1.48 ± 0.04 ^1^
5% (*v*/*v*) BP	-	-	1.05 ± 0.03 ^1^
0.5 μg mL^−1^ MMC	142.5 ± 23.5	162.5 ± 15.0	1.49 ± 0.02 ^1^
0.5% (*v*/*v*) BP + MMC (0.5 μg mL^−1^)	87.0 ± 9.0 ^a^	97.0 ± 7.0 ^a^	1.37 ± 0.02 ^1,a^
1% (*v*/*v*) BP + MMC (0.5 μg mL^−1^)	146.0 ± 2.0	160.5 ± 0.5	1.32 ± 0.14 ^1,a^
2% (*v*/*v*) BP + MMC (0.5 μg mL^−1^)	146.0 ± 0.0	162.0 ± 5.0	1.19 ± 0.01 ^1,a^
5% (*v*/*v*) BP + MMC (0.5 μg mL^−1^)	-	-	1.07 ± 0.05 ^1,a^

BNMN: micronucleated binucleated cells; MN: micronuclei; CBPI: cytokinesis block proliferation Index; BP: *Betula pendula* product containing aqueous leaf extract and lemon juice; MMC: mitomycin-C; MF (‰) ± se: mean frequencies (‰) ± standard error; MN were scored in 2000 binucleated lymphocytes per experimental point. ^1^ Significant difference compared to control at *p* < 0.001. ^a^ Significant difference compared to MMC at *p* < 0.001; G-test for BNMN and MN; *χ^2^* for CBPI.

**Table 3:** Compounds identified by UHPLC-MS analysis in BE and BP.

[M-H]^-^ (*m*/*z*)	Proposed Compound	BE	%A_BE_	BP	%A_BP_
285.2	Sakuranetin	√	11.6	√	1.0
463.2	Quercetin-3-*O*-galactoside (Hyperoside)	√	8.9	√	2.0
463.2	Quercetin-3-*O*-glucoside (Isoquercetin)	√	44.0	√	20.6
477.2	Quercetin-*O*-hexuronide	√	23.2	√	9.5
301.1	Quercetin	√	12.3	√	13.5
337.2	5-p-Coumaroylquinic acid			√	1.7
289.2	Catechin			√	1.8
433.10	Quercetin-3-*O*-arabinoside			√	3.3
431.3	Apigen-8-*C*-hexoside (Vitexin)			√	26.2
593.3	Isosakuranetin-7-*O*-rutinoside (Didymin)			√	1.2
461.3	Diosmetin-6-*C*-hexoside			√	4.1
461.3	Hispidulin-7-*O*-hexoside			√	1.1
479.2	Vanillic acid derivative			√	10.0
595.3	Eriodictoyl-7-*O*-rutinoside (Eriocitrin)			√	1.2
609.2	Hesperetin-7-*O*-rutinoside (Hesperidin)			√	1.4
579.3	Naringenin-7-*O*-rutinoside (Narirutin)			√	1.4

**Table 4:** Concentrations of elements in BE and BP.

Element (mg L^−1^)	BE	BP
**Mg**	0.68	18.9
**Al**	2.95	0.29
**Si**	21.0	4.38
**K**	4.33	76.4
**Ca**	1.47	5.47
**P**	3740	20.1
**Mn**	0.27	4.78
**Fe**	0.14	0.08
**Cu**	0.83	0.85
**Zn**	0.16	1.49

## Data Availability

Data are contained within the article.
